# Cutaneous Larva Migrans Infestation Over Buttocks and Perineal Region: A Case Series of Five Toddlers From Sri Lanka and Literature Review

**DOI:** 10.7759/cureus.11335

**Published:** 2020-11-05

**Authors:** Vijayakumary Thadchanamoorthy, Kavinda Dayasiri

**Affiliations:** 1 Clinical Sciences, Faculty of Health Care Sciences, Eastern University, Batticaloa, LKA; 2 Pediatrics, Base Hospital, Mahaoya, LKA

**Keywords:** cutaneous larva migrans, albendazole, buttocks, perineal region

## Abstract

Cutaneous larva migrans (CLM) is a cutaneous infestation caused by a hookworm larva. We report five toddlers who presented to the pediatric clinic with characteristic cutaneous lesions of CLM over the buttock and perianal region over periods of variable duration. Lesions of four children were typical and linear and one child had an atypical lesion. All were diagnosed as cutaneous larva migrans based on clinical history and examination. Complete recovery in all five children was achieved following treatment with oral albendazole.

## Introduction

Cutaneous larva migrans is cutaneous dermatitis caused by invasion and migration of parasitic larva of hookworms, including Ancylostoma duodenale and Necator americanus [1]. It has been reported among travelers and identified to be more common in tropical and subtropical countries [2]. The risk factors which facilitate cutaneous larva migrans (CLM) infection include poor sanitation, poor hygiene, and overcrowding. As it creeps and forms itchy erythematous linear tracts, it is also known as a creeping eruption. The diagnosis is mainly clinical based on the appearance of cutaneous tracts and treatment is usually with either albendazole or ivermectin depending on the availability of drugs [3]. We report five toddlers who presented with characteristic lesion over the buttocks and perianal region for the variable duration and the lesions were clinically compatible with CLM.

## Case presentation

Case 1

**Figure 1:**
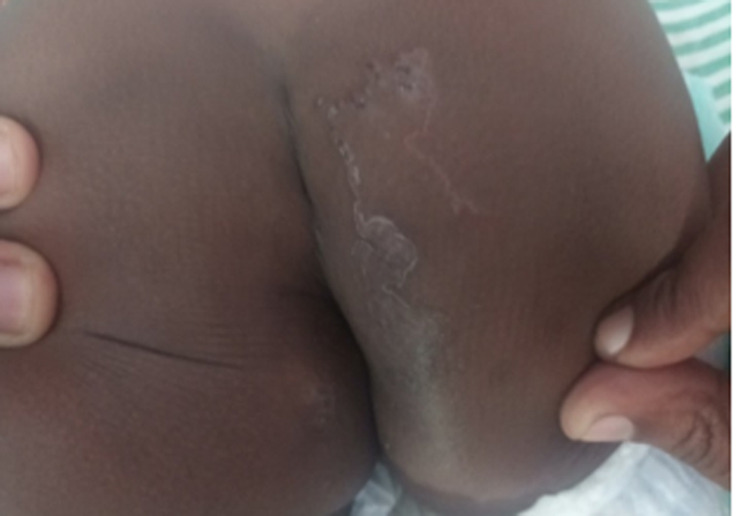
Linear itchy lesions over the buttock

A two-year-old female child presented to the dermatologic clinic with a history of pruritic lesions in the buttock for a one-month duration. The lesions initially appeared as papules, then migrated slowly and reached 10 cm in size. There was no past or family history of similar illness or any other comorbid illness. Her development and vaccination history were age-appropriate. The family had poor socioeconomic conditions and had several pets at home. The child had been playing in the soil frequently without underpants. Parents’ education was also poor. The child had been initially treated with antifungal and hydrocortisone by the general practitioner with no success. Examination revealed a linear, itchy tract measuring 10 cm and a lesion extended into the anus (Figure 1). Other systems examination was unremarkable. Her full blood count and C-reactive protein were within normal limits and there was no eosinophilia. She was clinically diagnosed as having CLM and treated with antihistamine and oral albendazole for three weeks. A complete recovery was observed after one month.

Case 2

**Figure 2:**
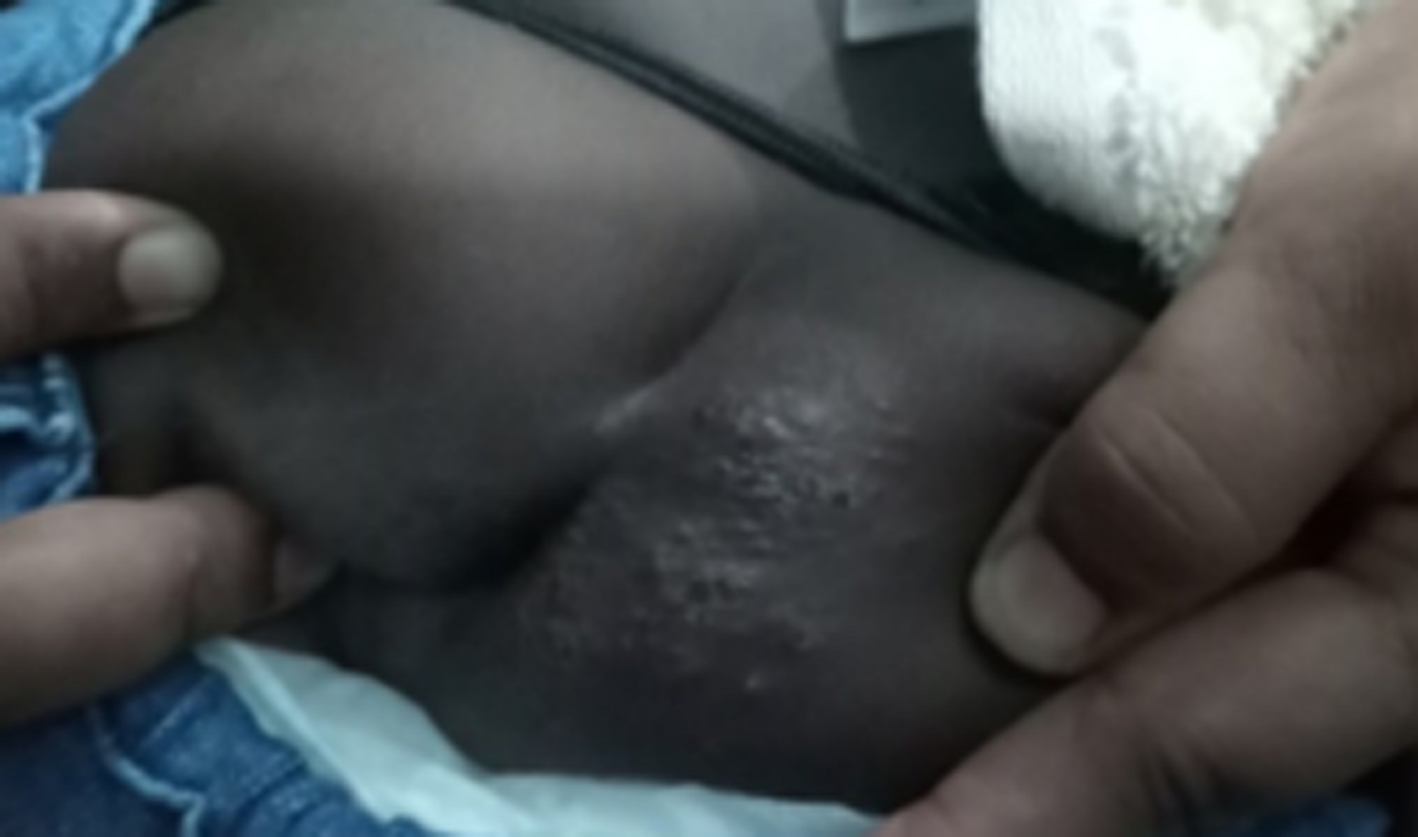
Coarse infected lesions in the perineal region

A one-year eleven-month-old female child presented to the pediatric clinic with irritability and poor sleep for a one-month duration. In addition, the child had an itchy skin lesion in the buttock which was a linear tract initially, and then it had become coarse and infected following scratching. The child also had a pruritic anus. She was treated with cloxacillin and chlorpheniramine oral suspensions for three weeks by the general practitioner with no success. Her development and immunization were age-appropriate. The child was frequently playing in the soil with her pet dog, which was vaccinated but not dewormed. Her mother also had a similar lesion on foot and it had disappeared spontaneously. Physical examination revealed a papular lesion with the underlying tract moving toward the anus and had been secondarily infected (Figure 2). Basic blood investigations were normal except for mild neutrophil leukocytosis (WBC - 13x103 cumm, neutrophil - 76%). The appearance of the lesion was compatible with CLM. She was treated with oral albendazole for three weeks and oral antibiotics for one week. Complete recovery of skin lesions was observed following treatment. 

Case 3

**Figure 3:**
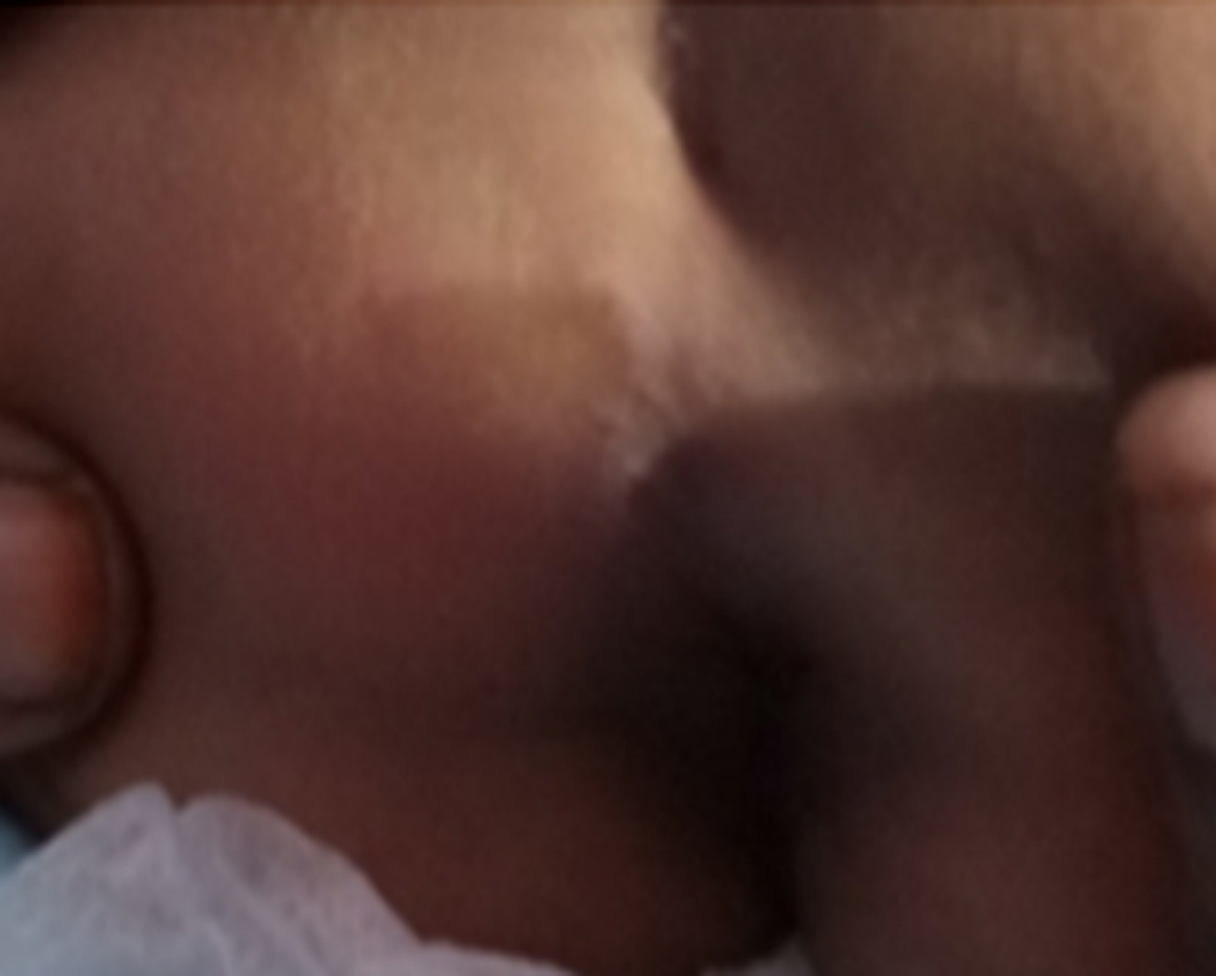
Linear lesion extending to the anus

A two-year six-month-old male child from very low socioeconomic conditions was referred to a nutritional clinic as the child had severe failure to thrive. He had been playing in the soil where plenty of dogs moved around and were defecating. His development and immunization were age-appropriate. Diet was inadequate both qualitatively and quantitatively. Examination revealed a linear, itchy, erythematous lesion in the perineal region (Figure 3) in addition to weight loss. The lesion was 8 cm in size. Blood investigations were normal except eosinophilia (WBC - 7.8x103 cumm, neutrophils - 40%, lymphocytes - 30%, eosinophils - 8%). The lesion was diagnosed as cutaneous larva migrans based on morphology and treated with oral albendazole for two weeks to achieve complete recovery. He was referred to the nutritional rehabilitation unit for follow-up.

Case 4

**Figure 4:**
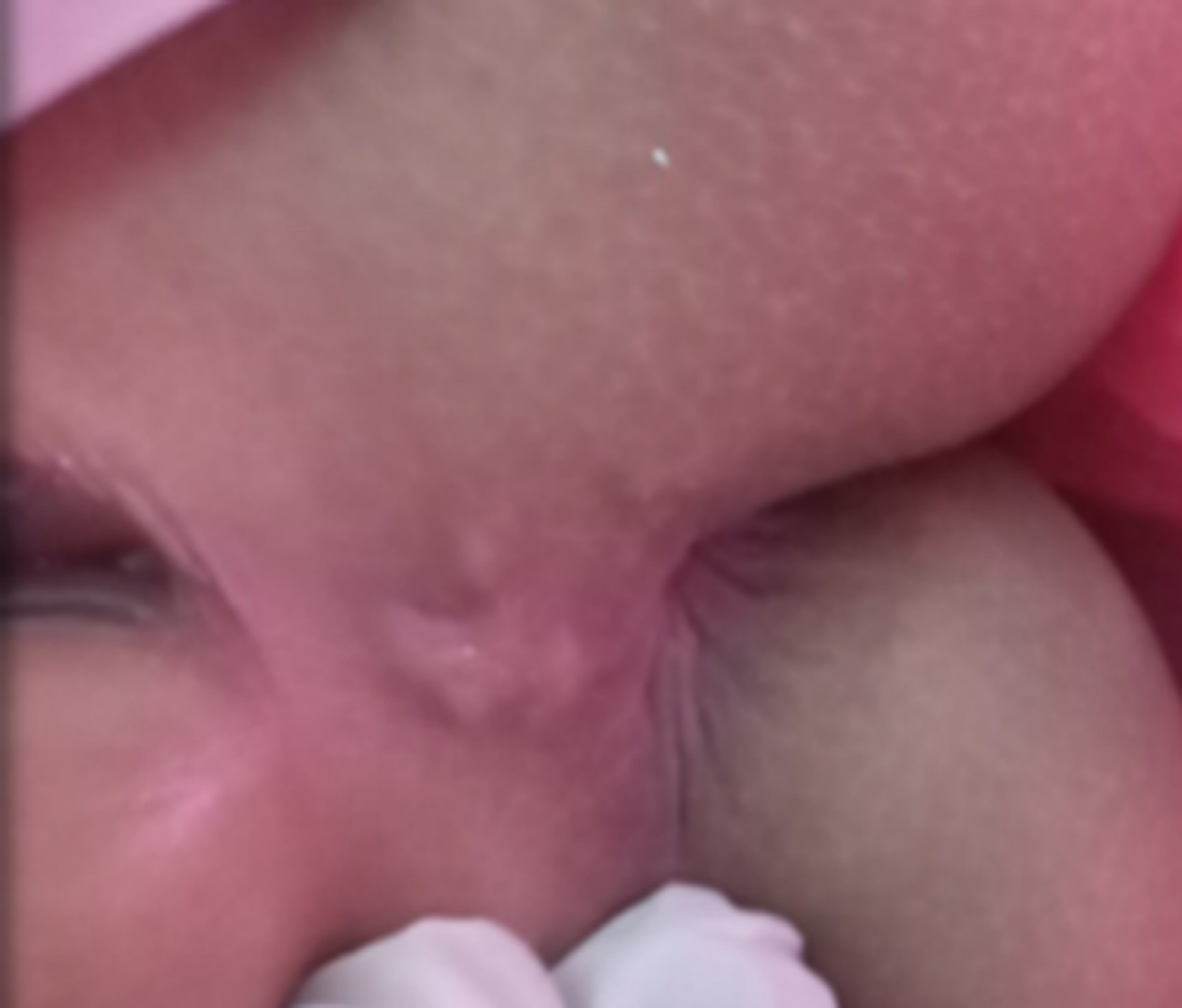
Lesions in fourchette

A three-year-old female child was referred by the medical officer of health suspecting possible child abuse at the routine examination in Well-baby clinic as the child had a lesion in the perineal region. The child was brought up by an educated family and there was no history of child abuse in detail inquiry, but the child has cared for in the daycare center where children were playing in the soil. Both parents were government employees. She had a pruritic lesion in the perineal area for three weeks, and the lesion was initially treated by a general practitioner as scabies with 5% permethrin local application and oral antihistamine without success. Her development and vaccination were up to date. Examination showed a well-grown child with a lesion in the fourchette area, sized 4 cm, itchy, plaque, and erythematous (Figure 4). Other system review and examination were clinically normal. Blood workup had been within normal limits. The diagnosis was confirmed as CLM based on the history and morphology of lesions, and she was treated with oral albendazole for 10 days with success.

Case 5

**Figure 5:**
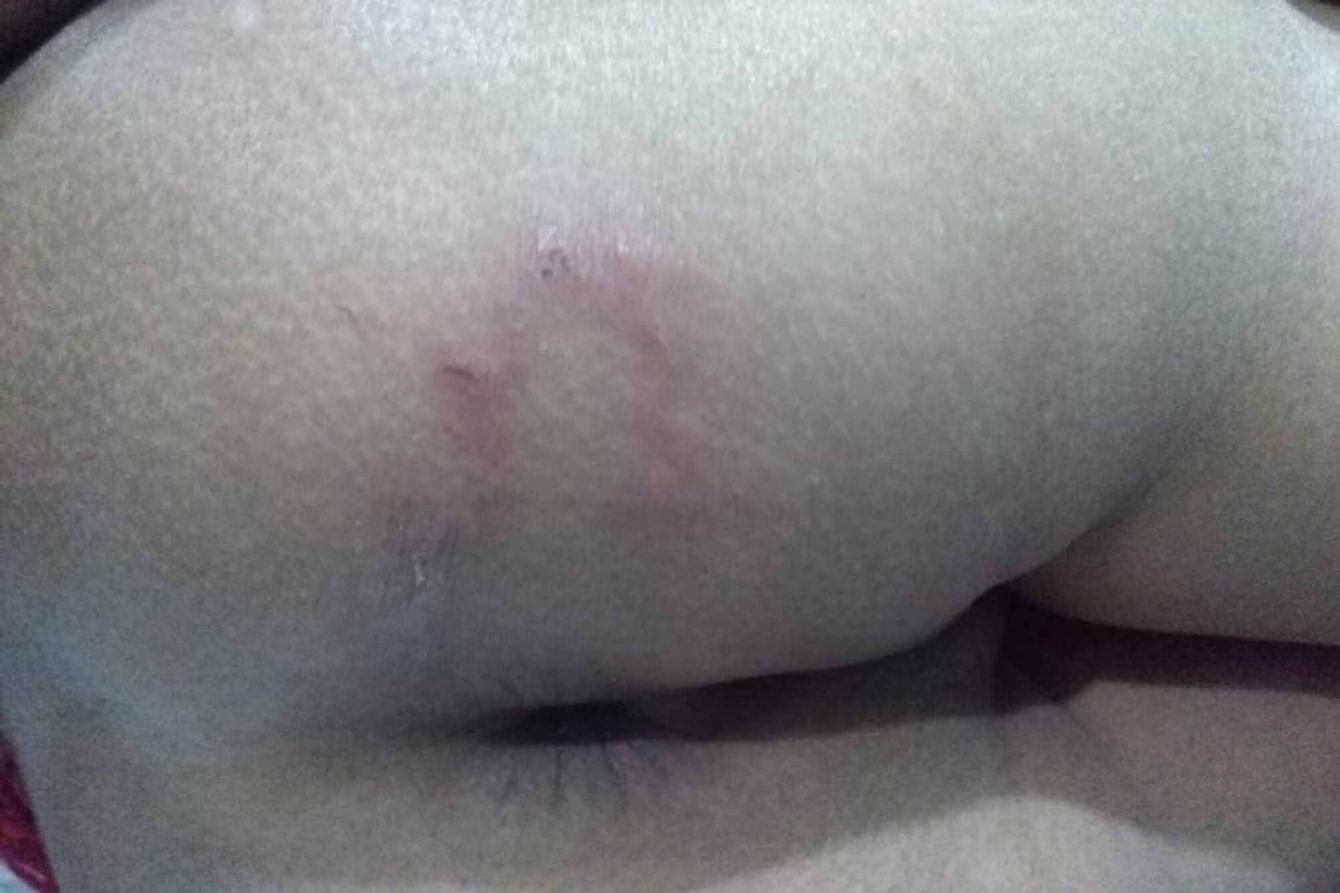
Curvilinear lesion in the buttock

A three-year-old female child was brought to the pediatric clinic having an itchy lesion in the buttock for a month. It had been moving in a linear line and disturbed the sleep. She had diplegic cerebral palsy and developmental delay and started to walk recently. Since then, she was playing with cats all the time and sitting in the soil without pants. Parents were government employees and the child had been cared for by the grandparents. The child was treated initially with miconazole cream as for tinea corporis by the outpatient department with no improvement. Her immunization was up to date. Physical examination revealed an erythematous and curvilinear lesion, sized 10-12 cm in length, and pruritic (Figure 5). Other system examination had been normal. Her basic blood investigations were within normal limits. The condition was diagnosed as cutaneous larva migrans and treated with oral albendazole for 14 days with complete improvement.

## Discussion

Cutaneous larva migrans is a neglected parasitic infestation in mostly unprivileged communities in unindustrialized countries [4,5]. It is a skin infestation caused by larvae mostly from animal nematodes, and rarely from insects [3]. The CLM is also known as creeping eruption, sand worms, plumber’s itch, duck hunter’s itch, and creeping verminous dermatitis [6]. It is more common in warmer climate countries and associated with poor socioeconomic condition [2]. A study from South Asia showed that it was more common in coastal areas which were a suitable environment for the infection to exist [3]. Four of five reported children had their residence in rural area and also had poor socioeconomic conditions. Children were playing in the soil without dresses and in underpants and also stray dogs and pets moved around the environment. Further, the villagers did not have knowledge regarding the infection and importance of deworming their domestic pets. Currently, there is no routine deworming program available in Sri Lanka.

Children acquire the disease when they are walking or playing in the soil contaminated with stools containing CLM. The larva penetrates and migrates the skin using enzymes, including proteases and hyaluronidase [7]. When the child sits and plays, the larvae enter through the dependent parts including buttock, hands, feet, and legs [7] and creep in the epidermis at the rate of 3 mm/day [6]. The incubation period is usually from one to six days. It forms a linear tract which might be erythematous and itchy. Larvae survive in the burrow for six to eight weeks and rarely up to two years [6]. As all five toddlers had been playing while sitting, they had the lesions in the buttock.

The symptoms and signs vary from typical sepernginous lesion to non-specific dermatitis. Usually it is a single larva tract, nevertheless multiple larvae might be active and form disorganized loops and tortuous tracks [1]. Possibly, severe infestation might cause hundreds of tracts. The lesions are intensely itchy, sometimes might produce burning sensation. Further itchiness might causes sleep disturbances and insomnia, and lesions can be complicated by secondary bacterial infections [3]. All reported children had a single tract, but one child had secondary infection and tract was not well defined due to scratching.

The differential diagnosis of CLM includes allergic contact dermatitis, urticarial factitia, pyodermas, scabies, cutaneous bacterial and fungal infections, photodermatitis and erythema chronicum migrans [8,9]. Diagnosis is made usually by clinical examination of lesions supported by history. The diagnosis may be missed due to its atypical appearance. Blood investigations might reveal secondary infection, eosinophilia, and high serum IgE (immunoglobulin E) [7]. Epiluminescence microscopy is an effective non-invasive method for detecting larvae [10], but is rarely necessary. Basic blood investigations had been done in all reported children; one child had eosinophilia and the other child had evidence of neutrophil leukocytosis. Investigations of other children were normal.

There are various treatment options available including topical and systemic treatment. Tropical treatment with thiabendazole needs repeated application for long duration. Also, it is associated gastrointestinal disturbances, and therefore it is rarely used [3]. Albendazole has been used since 1982 for CLM with success and considered to be the drug of choice for CLM. The appropriate dose is 400-800 mg/kg/day for seven days. Albendazole kills all stages of CLM although the mechanism of action is unknown. Further, it has minimal side-effects and is more potent [11,12]. The exact mechanism of action is not known, but it is thought to act by reducing or blocking the uptake of glucose, thus, in turn, decreasing or ceasing production of adenosine triphosphate (ATP). All five of these reported patients responded to oral albendazole for a variable duration, and no recurrences were observed. The other anthelminthic medication used has been ivermectin with the single dose of 150-200 mg/kg/day, which eradicates the parasite with negligible side effects [13]. The outcome of treatment was excellent, and no permanent scars were observed in any of the reported children.

Overall prevention of CLM is by provision of health education to public regarding personal hygiene and avoiding contact with pets to control CLM. Deworming the domestic and stray dogs and cats are also an effective method of prevention of CLM.

## Conclusions

This case series describes five toddlers with typical CLM lesions over the buttock and perineal region. CLM can be diagnosed clinically by its typical linear and itchy cutaneous tracts. Treatment with either albendazole or ivermectin leads to a complete cure.

## References

[1] Jelinek T, Maiwald H, Nothdurft HD, Löscher T (1994). Cutaneous larva migrans in travelers: synopsis of histories, symptoms, and treatment of 98 patients. Clin Infect Dis.

[2] Yavuzer K, Ak M, Karadag AS (2010). A case report of cutaneous larva migrans. Eurasian J Med.

[3] Karthikeyan K, Thappa DM (2002). Cutaneous larva migrans. Indian J Dermatol Venereol Leprol.

[4] Heukelbach J, Feldmeier H (2008). Epidemiological and clinical characteristics of hookworm-related cutaneous larva migrans. Lancet Infect Dis.

[5] Heukelbach J, Mencke N, Feldmeier H (2002). Cutaneous larva migrans and tungiasis: the challenge to control zoonotic ectoparasitoses associated with poverty. Trop Med Int Health.

[6] Padmavathy L, Rao LL (2005). Cutaneous larva migrans - a case report. Indian J Med Microbiol.

[7] Siddalingappa K, Murthy SC, Herakal K, Kusuma MR (2015). Cutaneous larva migrans in early infancy. Indian J Dermatol.

[8] Davies HD, Sakuls P Keystone JS (1993). Creeping eruption. A review of clinical presentation and management of 60 cases presenting to a tropical disease unit. Arch Dermatol.

[9] Albanese G, Venturi C, Galbiati G (2001). Treatment of larva migrans cutanea (creeping eruption): a comparison between albendazole and traditional therapy. Int J Dermatol.

[10] Elsner E, Thewes M, Worret WI (1997). Cutaneous larva migrans detected by epiluminescent microscopy. Acta Derm Venereol.

[11] Coulaud JP, Binet D, Voyer C, Samson C, Moreau G, Rossignol JF (1982). Treatment of the cutaneous larva migrans syndrome "Larbish" with albendazole. Bull Soc Pathol Exot Filiales.

[12] Jones SK, Reynolds NJ, Oliwiecki S, Harman RRM (1990). Oral albendazole for the treatment of cutaneous larvae migrans. Br J Dermatol.

[13] Kaur S, Jindal N, Sahu P, Jairath V, Jain VK (2015). Creeping eruption on the move: a case series from Northern India. Indian J Dermatol.

